# Supplementary material for: Coupling between tolerance and resistance for two related Eimeria parasite species
Source: Ecol Evol. 2020 Nov 12;10(24):13938–48. doi: 10.1002/ece3.6986 (PMC7771152; doi:10.1002/ece3.6986)
Supplement: Supplementary file 1 — Appendix S1 [file ECE3-10-13938-s001.pdf]

	Mouse strain (species)								
**Batch**	SCHUNT (Mmd)	STRA (Mmd)	SCHUNTx STRA (Mmd)	STRAx BUSNA (Mmd-Mmm)	SCHUNTx PWD (Mmd-Mmm)	PWDx BUSNA (Mmd)	BUSNA (Mmm)	PWD (Mmm)	***Eimeria* isolate (species)**
**B1**	3	4					2	3	Brandenburg64 (*E. ferrisi*)
**B2**	4	4					5	3	Brandenburg64 (*E. ferrisi*)
**B3**	3	3	2	2	3	3	3	3	Brandenburg64 (*E. ferrisi*)
	3	3	3	4	3	3	3	3	Brandenburg88 (*E. falciformis*)
**B4**	4	4	4	6	5	6	4	4	Brandenburg64 (*E. ferrisi*)
	3	4	5	4	3	4	4	4	Brandenburg88 (*E. falciformis*)
