# Supplementary figures and images for: Coupling between tolerance and resistance for two related Eimeria parasite species
Source: Ecol Evol. 2020 Nov 12;10(24):13938–48. doi: 10.1002/ece3.6986 (PMC7771152; doi:10.1002/ece3.6986)

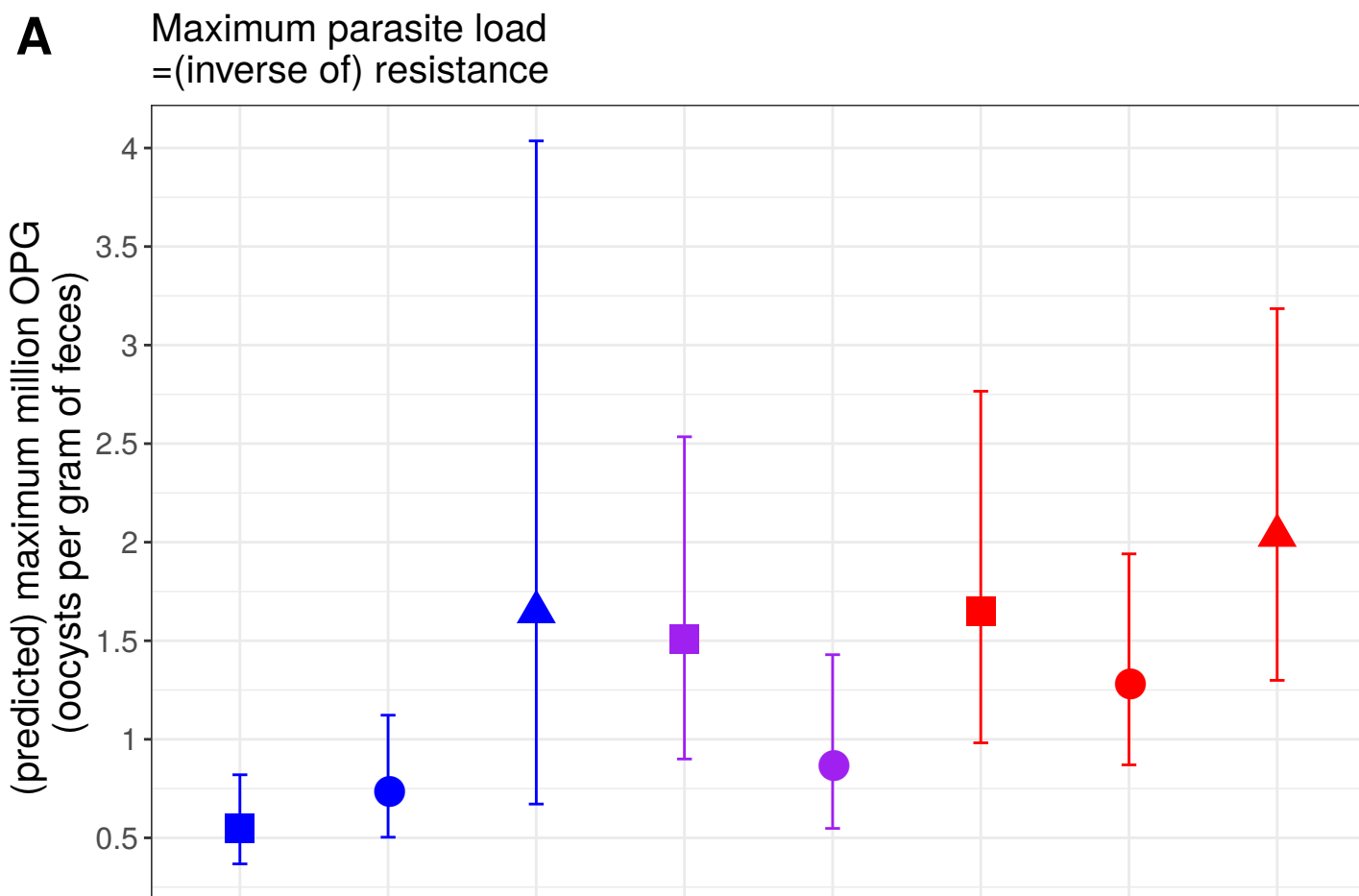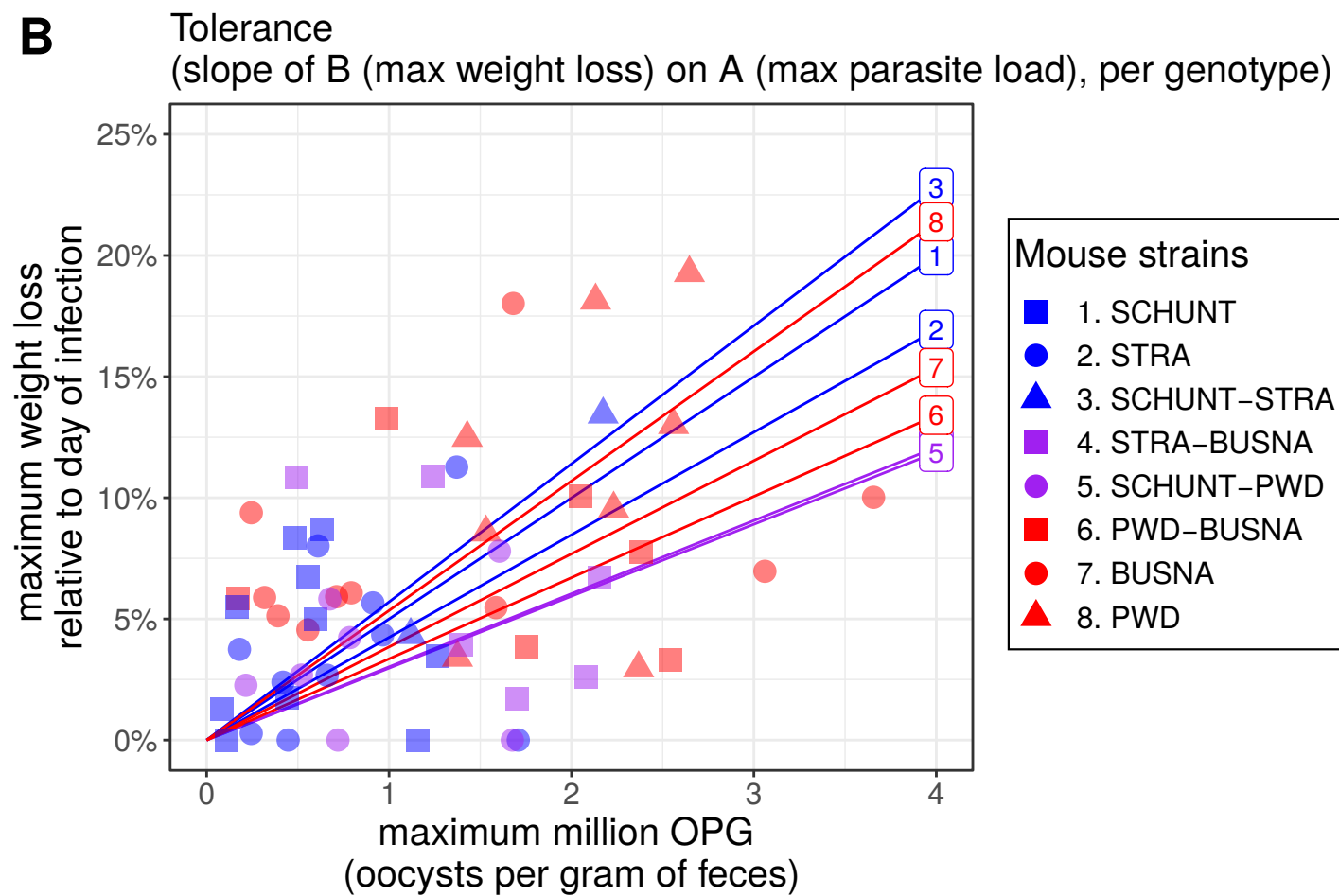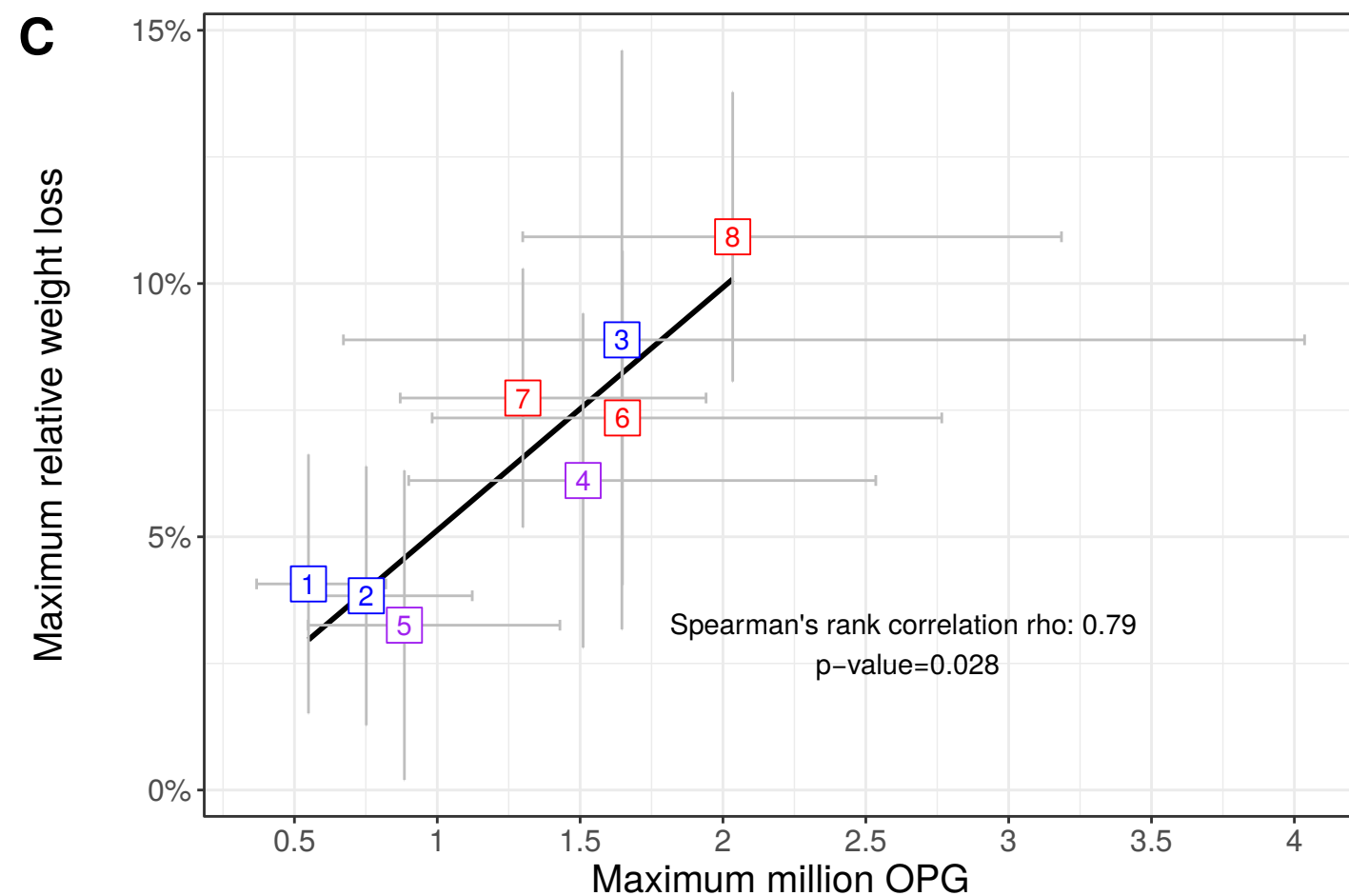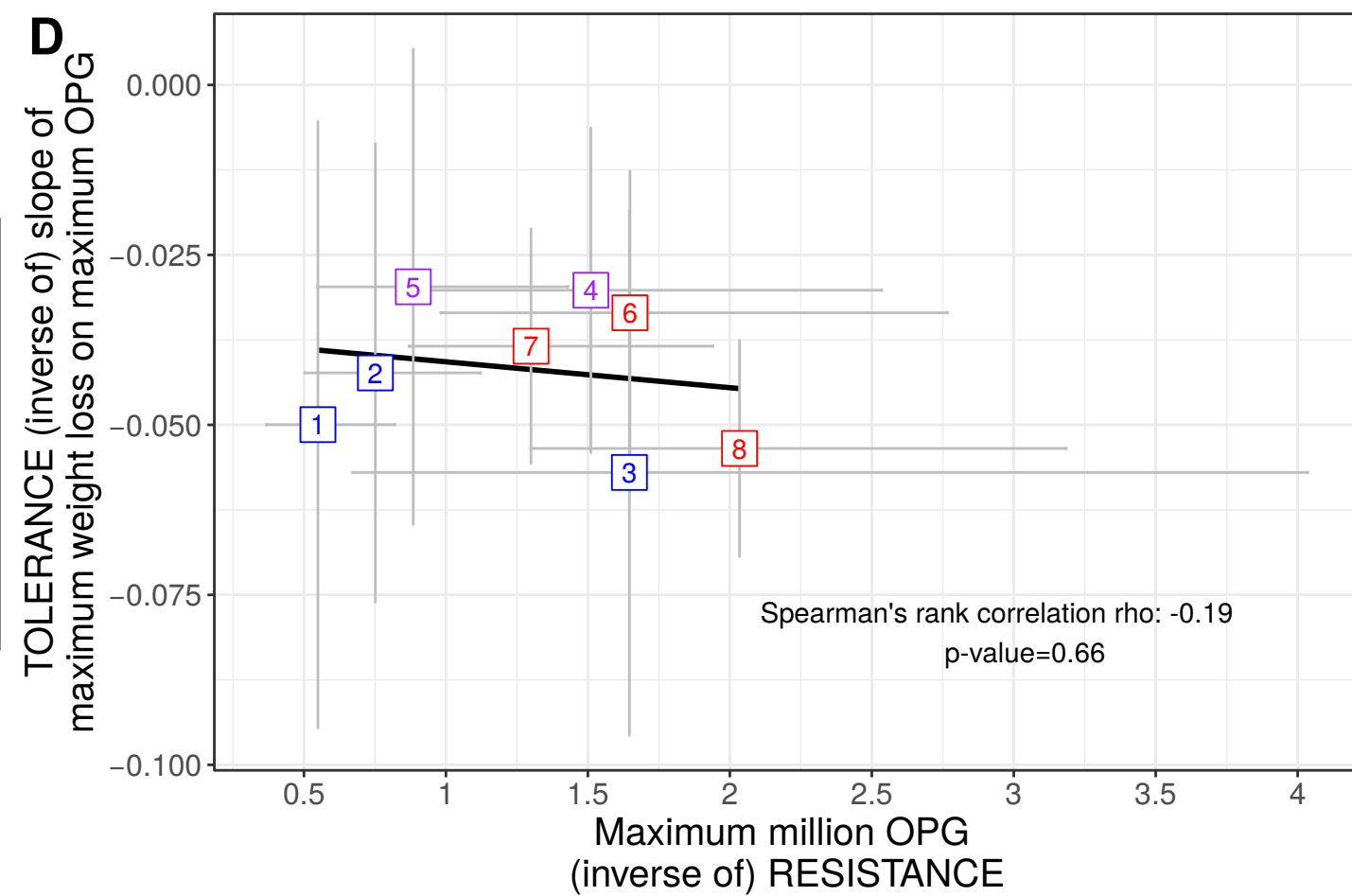

Supplement: Supplementary file 2 — Appendix S2 [file ECE3-10-13938-s002.pdf]

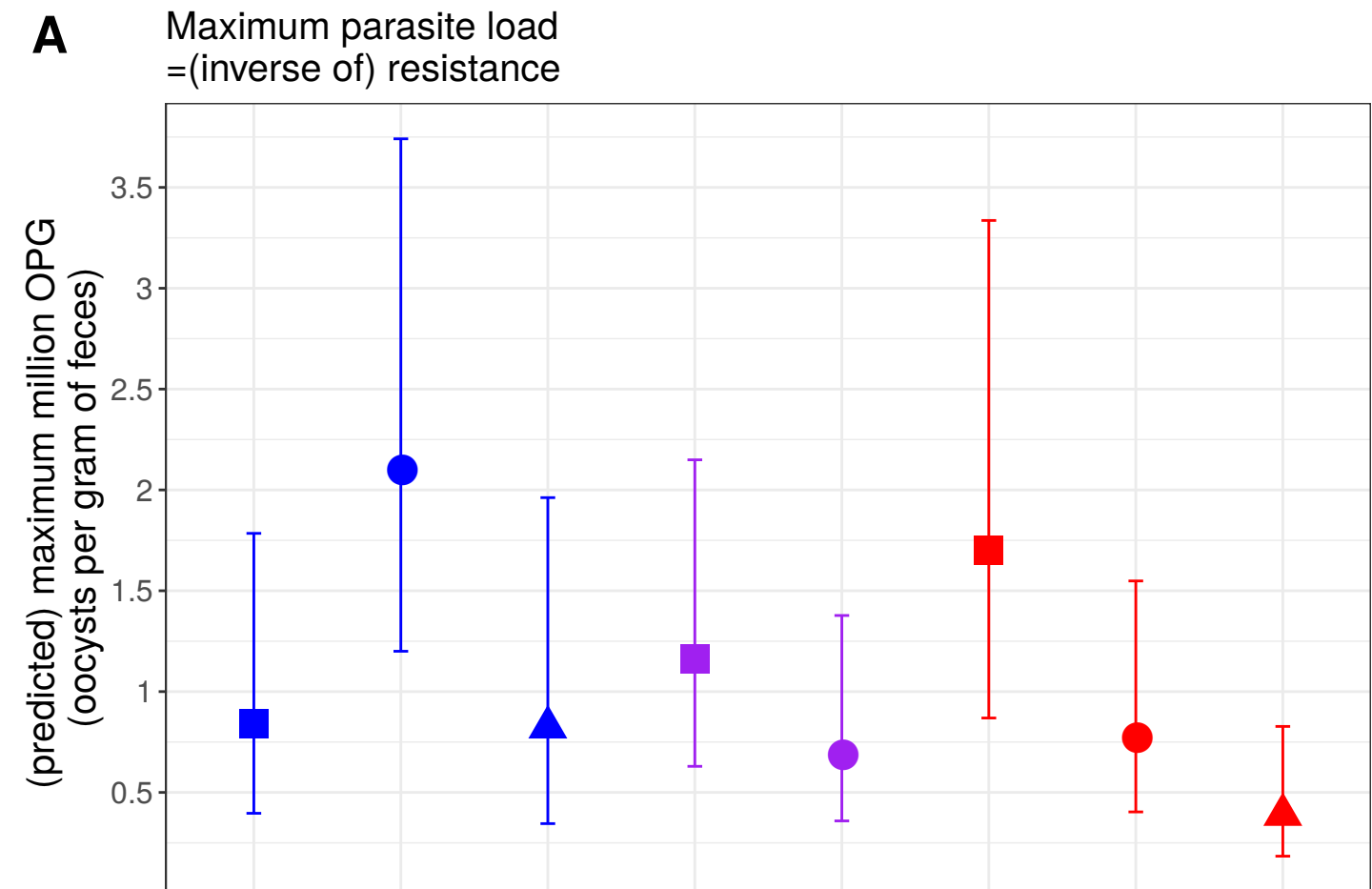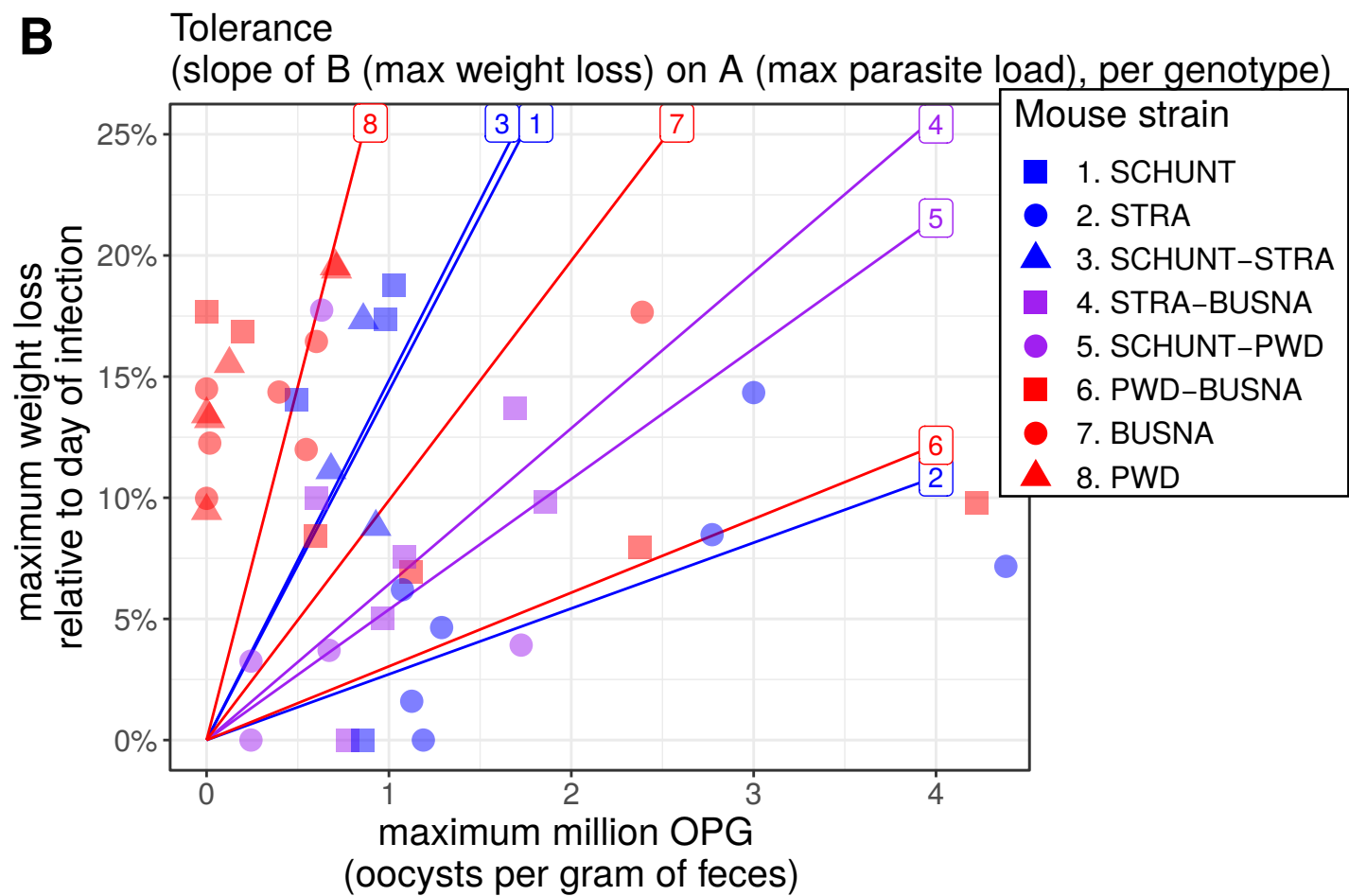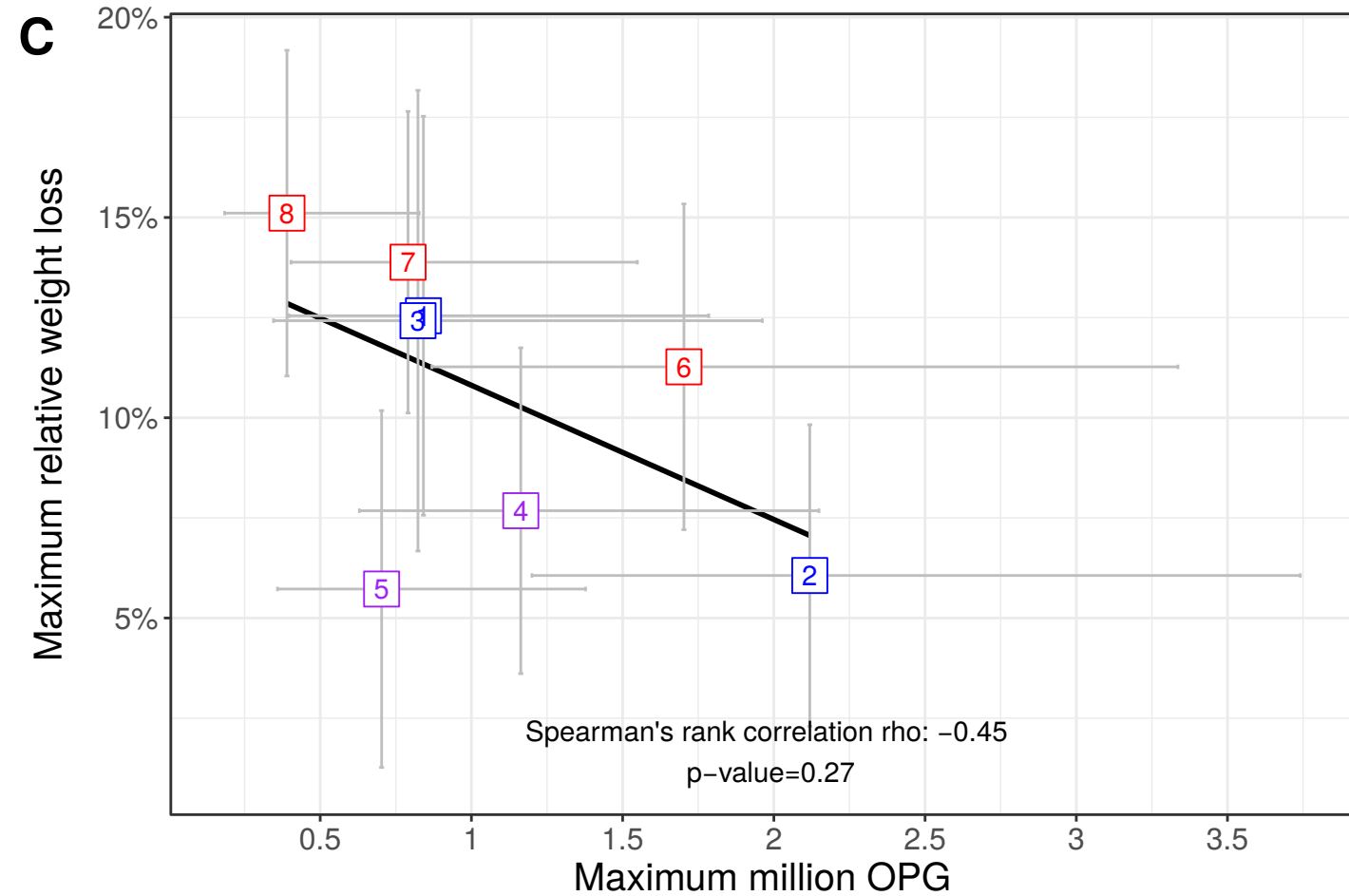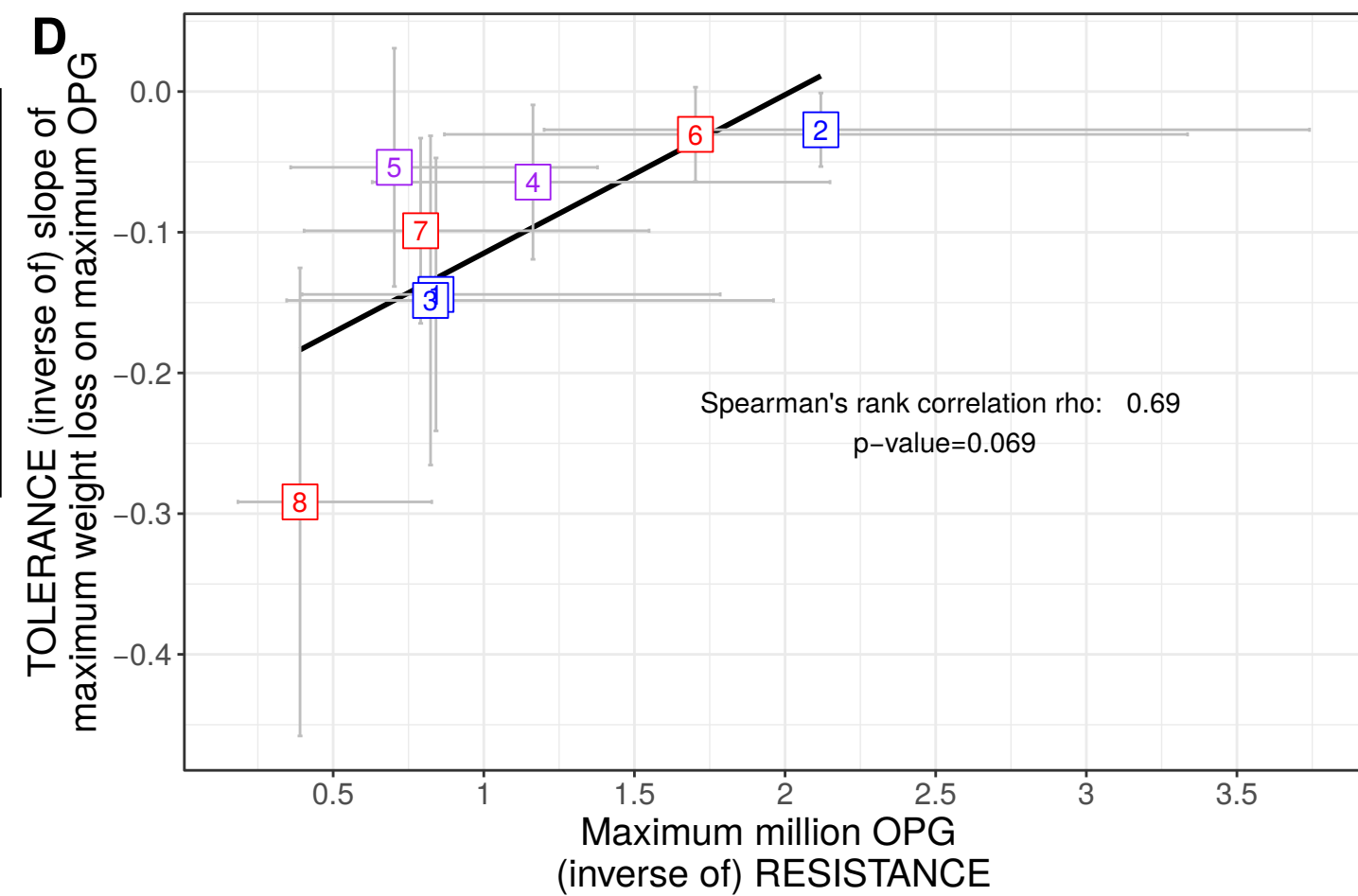

Supplement: Supplementary file 3 — Appendix S3 [file ECE3-10-13938-s003.pdf]
