# Supplementary material for: Coupling between tolerance and resistance for two related Eimeria parasite species
Source: Ecol Evol. 2020 Nov 12;10(24):13938–48. doi: 10.1002/ece3.6986 (PMC7771152; doi:10.1002/ece3.6986)
Supplement: Supplementary file 4 — Supplementary Material [file ECE3-10-13938-s004.docx]

**Appendix S1. Chronology of experimental infections.**

**Appendix S2. No indication of resistance–tolerance coupling for *E. ferrisi* isolate Brandenburg64 in the conservative data set.** Colors represent mouse subspecies (blue: *M. m. domesticus*, red: *M. m. musculus*, purple: Mmd-Mmm). Left side: comparison of maximum oocysts per gram of feces used as a proxy for (inverse of) resistance (A) and tolerance (B) between mouse groups estimated by the slope of the linear regression with null intercept modeling maximum relative weight loss as a response of maximum oocysts per gram of feces, a steep slope corresponding to a low tolerance. Maximum number of OPG differs between mouse groups, but tolerance is similar. Right side: positive correlation between mean maximum oocysts per gram of feces and mean relative weight loss (C) and absence of correlation between maximum oocysts per gram of feces used as a proxy for (inverse of) resistance and tolerance (D); grey error bars represent 95% confidence intervals. Our results do not support coupling between resistance and tolerance *E. ferrisi* isolate Brandenburg64.

**Appendix S3. Coupling between resistance and tolerance for *E. falciformis* isolate Brandenburg88 in the conservative dataset.** Colors represent mouse subspecies (blue: *M. m. domesticus*, red: *M. m. musculus*, purple: Mmd-Mmm). Left side: comparison of maximum oocysts per gram of feces used as a proxy for (inverse of) resistance (A) and tolerance (B) between mouse groups estimated by the slope of the linear regression with null intercept modeling maximum relative weight loss as a response of maximum oocysts per gram of feces, a steep slope corresponding to a low tolerance. Maximum number of OPG and tolerance differ between mouse groups. Right side: nonsignificant negative correlation between mean maximum oocysts per gram of feces and mean relative weight loss (C) and strong positive correlation between maximum oocysts per gram of feces used as a proxy for inverse of resistance and tolerance (corresponding to a negative correlation between resistance and tolerance) (D); grey error bars represent 95% confidence intervals. Our results support coupling between resistance and tolerance *E. falciformis* isolate Brandenburg88.
